# A database for reproducible manipulation research: CapriDB – Capture, Print, Innovate

**DOI:** 10.1016/j.dib.2017.02.015

**Published:** 2017-02-12

**Authors:** Florian T. Pokorny, Yasemin Bekiroglu, Karl Pauwels, Judith Butepage, Clara Scherer, Danica Kragic

**Affiliations:** a KTH Royal Institute of Technology, Sweden; b ABB Corporate Research, Sweden

## Abstract

We present a novel approach and database which combines the inexpensive generation of 3D object models via monocular or RGB-D camera images with 3D printing and a state of the art object tracking algorithm. Unlike recent efforts towards the creation of 3D object databases for robotics, our approach does not require expensive and controlled 3D scanning setups and aims to enable anyone with a camera to scan, print and track complex objects for manipulation research. The proposed approach results in detailed textured mesh models whose 3D printed replicas provide close approximations of the originals. A key motivation for utilizing 3D printed objects is the ability to precisely control and vary object properties such as the size, material properties and mass distribution in the 3D printing process to obtain reproducible conditions for robotic manipulation research. We present CapriDB – an extensible database resulting from this approach containing initially 40 textured and 3D printable mesh models together with tracking features to facilitate the adoption of the proposed approach.

**Specifications table**

**Table:** 

Subject area	Computer Science
More specific subject area	Computer Vision, Robotics, Computer Graphics
Type of data	3D mesh models
How data was acquired	Monocular camera
Data format	Wavefront OBJ, MDL, JPEG
Experimental factors	Post-processing in order to result in a clean and fully specified model
Experimental features	3D model construction, post-processing
Data source location	The database and associated documentation is hosted at 〈http://www.csc.kth.se/capridb/〉
Data accessibility	Public: 〈http://www.csc.kth.se/capridb/〉

**Value of the data**

• The data includes 3D object models through an efficient and low-cost workflow to capture, print and track new objects.
• The data allows anyone to reproduce objects based on the provided 3D mesh models and to use these for robotic manipulation research in a controlled and comparable fashion.
• Rather than relying on the original objects the database makes use of 3D printing and we provide a specialized tracking solution as well as a low-cost approach to 3D scanning which does not rely on a specific scanning setup or object scale but only on a hand-held camera.
• We verify that the obtained object models can be 3D printed with texture and that the pose of these printed objects can be tracked successfully. We furthermore perform initial grasping experiments using the estimated poses of printed objects which are calculated using the mesh-models obtained from the original real-world objects.

## 1 Description of data

**Fig. 1:**
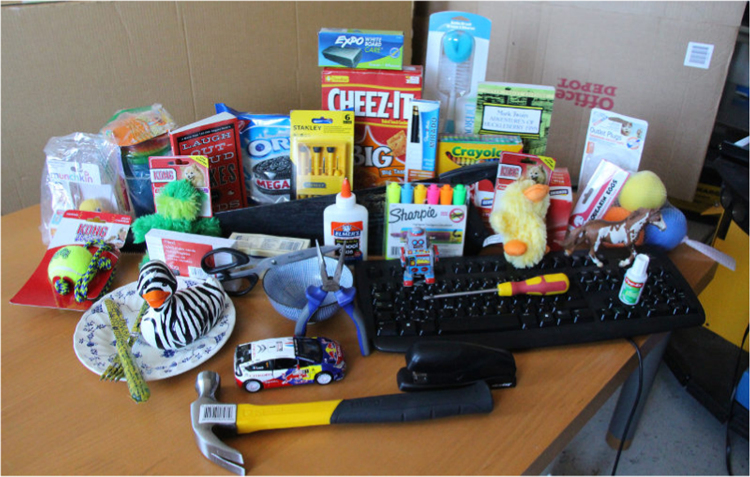
Initial set of 40 objects in the core database.

**Fig. 2:**
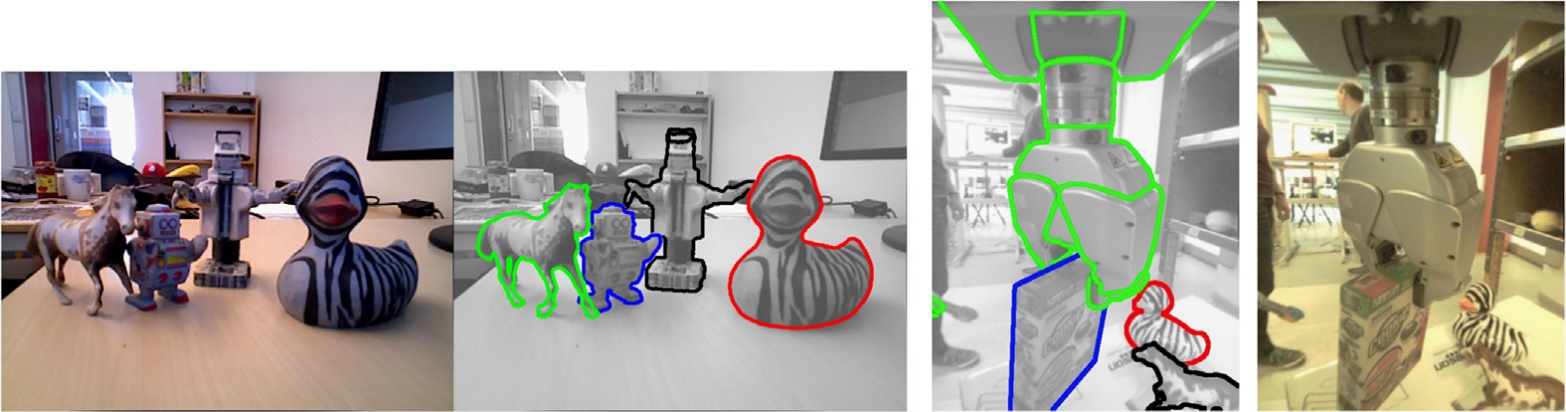
Left: Examples of 3D printed objects whose 3D textured model was acquired using the proposed methodology: A horse model, a toy robot, a rescaled PR2 robot and a toy duck. Middle: Pose tracking results of the printed objects based on textured models acquired on the originals. Right: Example tracking of printed objects based on the PR2 robot׳s arm camera.

**Fig. 3:**
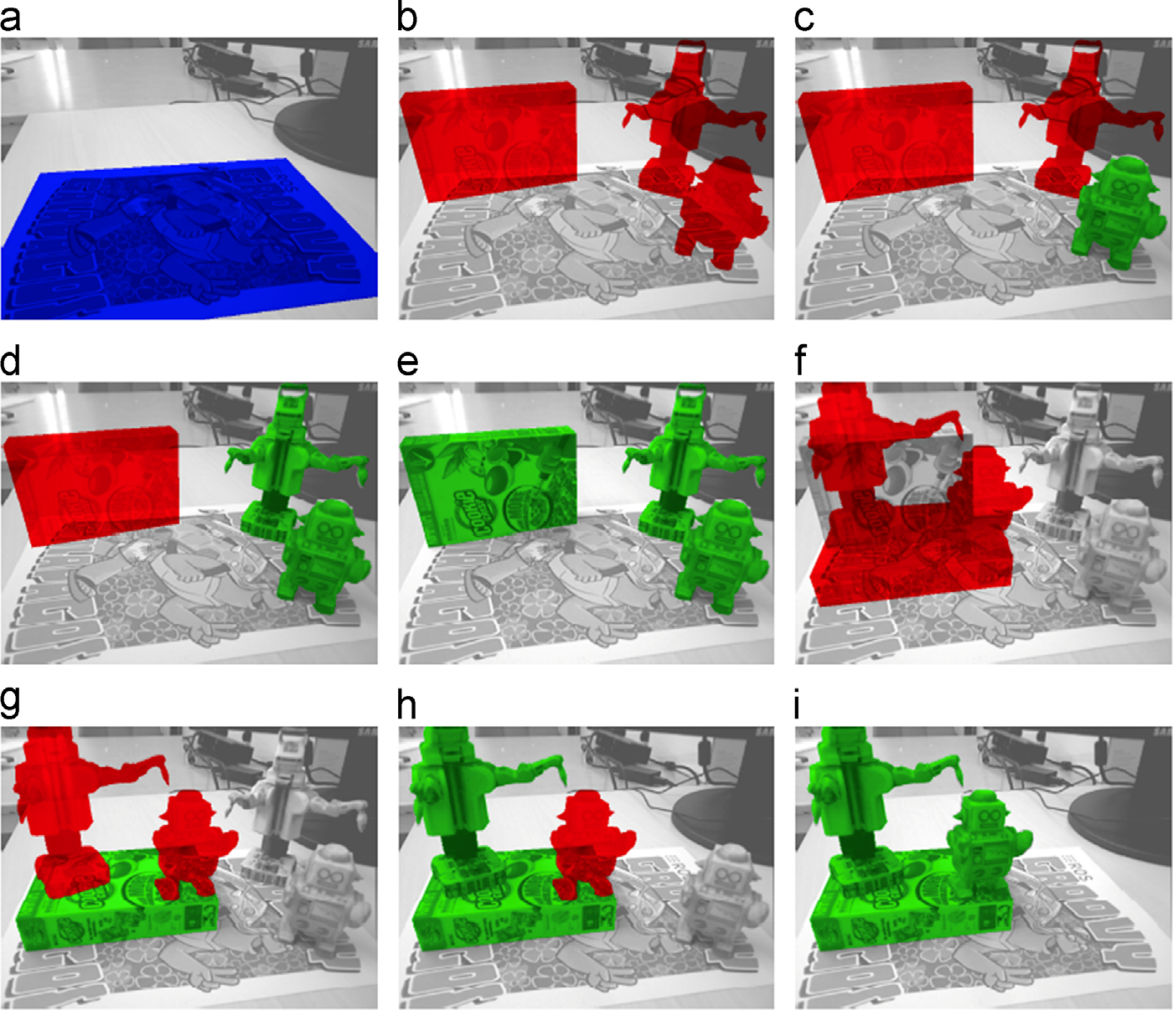
Example benchmarking using a pre-defined scene layout: (a) a marker is introduced in the scene, detected, and highlighted in blue. This marker provides a reference frame for the scene. (b) The desired object placement according to a pre-defined initial scene layout is highlighted in red. (c–e) One-by-one, the objects are placed in the scene and the color changes to green if their placement is sufficiently accurate. (f) A pre-defined target scene layout is highlighted in red. (g–i) The task is executed and the objects are moved to their target pose.

The initial release of CapriDB contains 40 watertight textured mesh models of the objects listed in Table 1 and depicted in Fig. 1. Mesh models are stored in Wavefront OBJ format, a mesh to texture mapping is provided in MDL format and an associated texture file is stored as a JPEG image for each object. The objects for the 2015 IEEE ICRA Amazon Picking Challenge are also included in the database. Table 1 lists the physical dimensions of these objects, their weight and original material as well as additional notes which will also be stored in CapriDB. In addition, the initial database release contains the original photos (approx. 40 per object) used to construct the mesh approximation in JPEG format. To facilitate performance evaluation on the applications of the database, we also include reference images (in JPEG) and associated tracking boundaries (overlaid JPEG based on object poses acquired from the tracker) for each object as in Fig. 2 to test and compare other tracking methodologies. Fig. 3 shows how the database and interactive tracking could be used for an example benchmarking approach using a pre-defined scene layout. The included scenes and object poses can be used as ground truth to set up a system using these object models and the tracker. More information about the tracker׳s accuracy can be found in [1].

## 2 Experimental design, materials and methods

The data is prepared using Autodesk123D catch services [2] based on approximately 40 pictures of each object from various angles. We place the objects on a textured background consisting of a collection of newspapers. The acquired 3D mesh models require post-processing in order to result in a clean and fully specified model. We use the software Meshlab [3] to remove parts of the mesh that belongs to the surroundings. As a last step we manually ensure that no holes exist in the objects using the software Blender [4].

### 2.1 Data capture and methodology

#### 2.1.1 Textured 3D model construction

**Fig. 4:**
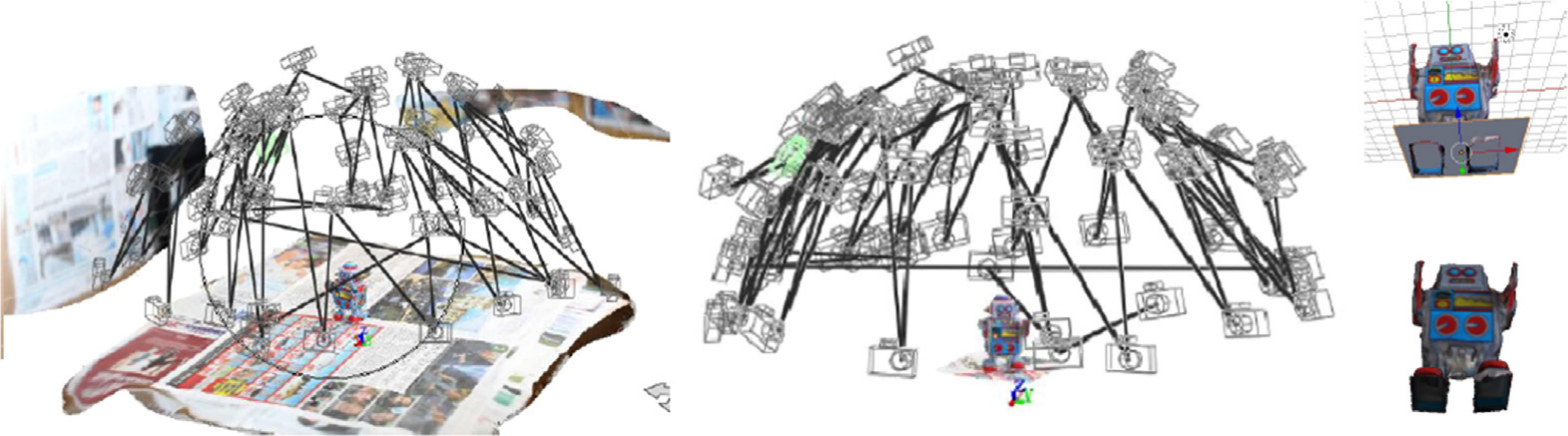
Left and middle figures: construction of the 3D model with Autodesk’s free 123D catch application [2]. The reconstructed camera poses and the central object pose is displayed. Rightmost figures: post-processing of the acquired textured mesh model, where the mesh is made watertight and surface areas not belonging to the object are removed.

While current grasp databases often rely on carefully calibrated specific capturing equipment, e.g., [5], our approach is to use a simple digital camera in conjunction with a freely available 3D reconstruction software to capture 3D objects. This approach has recently become possible due to the availability of high-quality 3D reconstruction software relying only on monocular images. To reconstruct a 3D model from a collection of photos, we utilize the web-based free Autodesk 123D catch service [2] using approximately 40 pictures of the object from various angles. To improve the quality of reconstruction, we place the objects on a textured background consisting of a collection of newspapers. Fig. 4 displays a partial screenshot of the software, illustrating the automatically reconstructed camera positions. The scanned object is visible in the center of this visualization.

#### 2.1.2 3D model post-processing

The acquired 3D mesh model requires post-processing in order to result in a clean and fully specified model. Detailed instructions regarding this process are available via http://csc.kth.se/capridb. Firstly, the metric dimensions of the model have to be specified in centimeters with the help of reference points for which we use the Autodesk 123D catch software [2]. As the initially obtained 3D mesh model contains not only the object but also some parts of the surrounding environment, such as the surface on which the object might rest, these extraneous parts of the extracted mesh need to be removed. We use the open source software Meshlab [3] for this purpose. Fig. 4 illustrates post-processing steps where areas that do not belong to the object are manually removed from the initial model. In the final manual processing step, holes in the mesh are closed. Holes arise, for example on the underside of the object, when the object rests on a planar surface when the photos are taken. For the hole filling, we used the open source 3D modeling software Blender [4], which also can be used for rotating and scaling the models as desired. Furthermore, we utilize a specific object pose tracker, which we describe later, to demonstrate that the pose of these models can be tracked. The tracker requires the dimensions of the mesh model to be provided in meters, in accordance with the ROS convention. Therefore, as a final post-processing step the models are scaled accordingly. After this processing step, we obtain a mesh model whose geometry is stored in Wavefront OBJ format, a mesh to texture mapping stored in MDL format as well as a texture file, which is stored as a JPEG image.

#### 2.1.3 3D printing textured objects

**Fig. 5:**
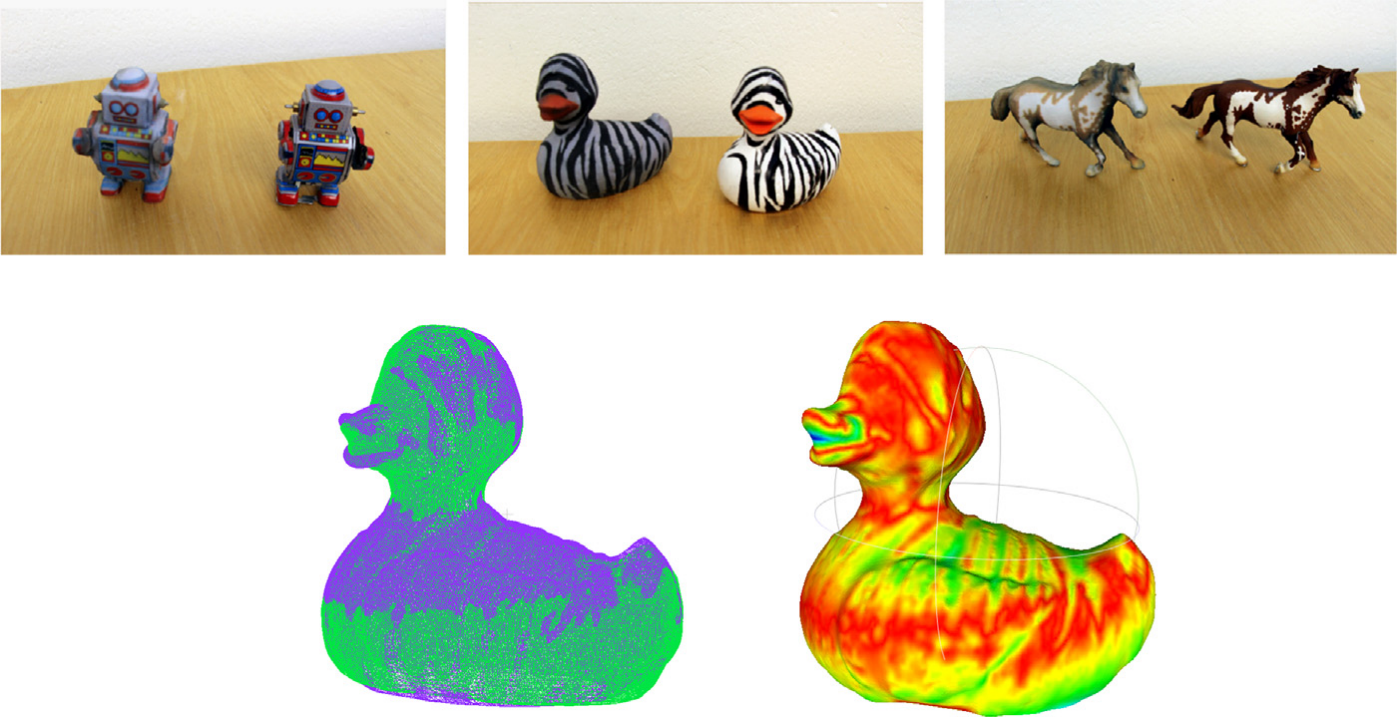
Top: side-by-side comparison of original models (right) and 3D printed objects (left). Bottom: overlay of re-scanned mesh models for the duck original and printed version aligned via ICP (left) and coloring of duck model by Hausdorff difference between printed and original model (right).

Our goal is to make manipulation objects widely accessible as 3D mesh models and in physical/graspable forms. The rapidly advancing field of 3D printing makes it possible to 3D print objects rather than having to obtain originals which may only be available locally. A large range of on-line services offer to print highly textured objects in color. This allows anyone to reproduce objects based on the provided 3D mesh models and to use these for robotic manipulation research. We have printed several objects (see Fig. 5) through the company iMaterialise [6]. Note that 3D printing also enables scaling objects as desired, and to vary the internal mass distribution by partially filling objects solidly. One can furthermore select a wide range of object materials. This opens up promising new possibilities to study frictional and dynamic behavior in robotic manipulation in a controlled fashion and independently of shape in future. Fig. 2, Fig. 5 display examples of printed objects, which we scanned and printed.

To test the quantitative difference between printed and original objects, we utilized a MakerBot Digitizer 3D scanner [7] to scan both the original and printed duck object with high accuracy. We then aligned the mesh models via the Iterative Closest Point Method (ICP), using the implementation provided in the CloudCompare software [8]. The bottom part of Fig. 5 displays an overlay of the vertices of the resulting aligned mesh models on the left. The two models have a root mean square distance of 0.92 mm and maximal distance of 4.12 mm in terms of Hausdorff distance computed on the approximately 70,000 points sampled from the meshes utilizing the Meshlab software [3]. The right part of the figure displays a visualization of the point-wise differences between the models obtained using Meshlab [3].

#### 2.1.4 Tracking and pose estimation

We use a state-of-the-art image-based object pose estimation method that uses sparse keypoints to detect, and dense motion and depth information to track the full six degrees-of-freedom pose in real-time [1], [9]. This method has been demonstrated to achieve high accuracy and robustness by exploiting the rich appearance and shape information provided by the models in our database. This pose estimation method is publicly available as a ROS module (SimTrack [10]). We provide a proof of concept validation of our proposed methodology by successfully detecting and tracking the pose of printed and original objects on the basis of the mesh models generated from the original objects.

**Fig. 6:**
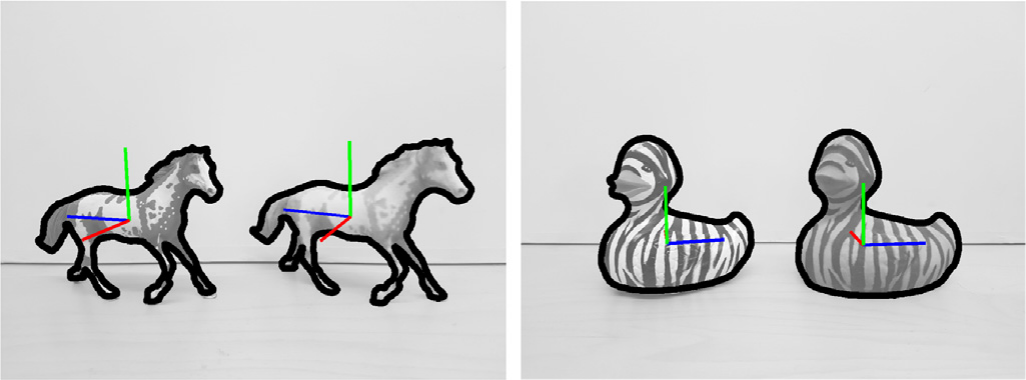
Side-by-side tracked poses and overlayed mesh boundaries from images of the horse and duck original and printed objects. Also the relative poses can be observed based on the attached frames (red=x, green=y, blue=z). (For interpretation of the references to color in this figure, the reader is referred to the web version of this article.)

**Fig. 7:**
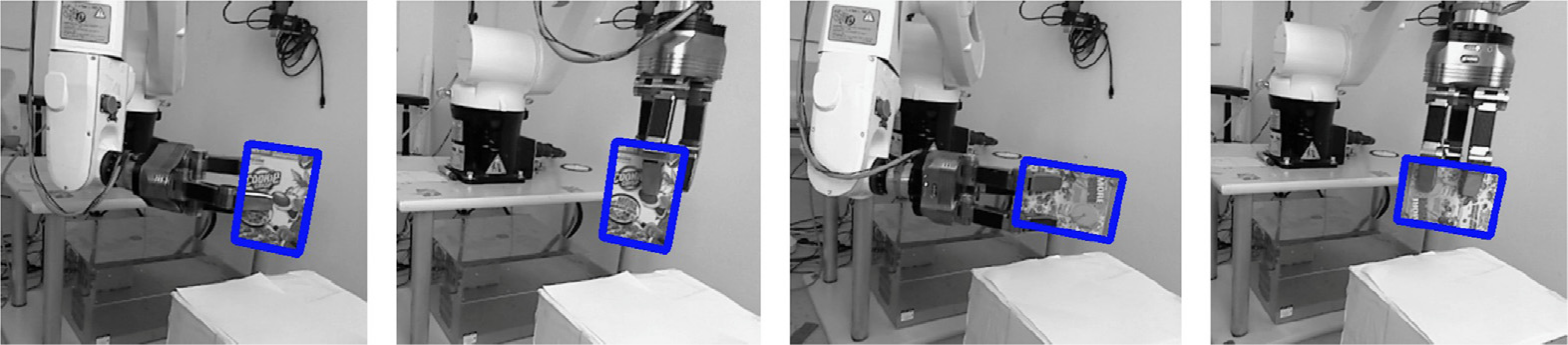
Grasping experiments with a printed object: side and top grasps by placing the wrist to a predefined distance from the object’s center along its vertical, and horizontal axis and closing the fingers.

Fig. 6 shows tracking results using horse and duck originals and printed objects placed side-by-side validating the performance of SimTrack [10]. Example tracking results with occlusions and multiple 3D printed objects are shown in Fig. 2. A PR2 robot׳s onboard arm camera is also used to track several 3D printed objects. Both RGB and RGB-D cameras can be used with this approach. Example grasping experiments with a Kuka arm, a Schunk Hand and a printed box based on the object poses estimated by the tracker which used the 3D model obtained from the real object can be seen in Fig. 7. These experiments indicate that the texture of the printed object matched the original texture sufficiently well, but results may vary for objects with limited texture structure. In this experiment, images from a Kinect sensor were used. The object can continuously be tracked during grasping and lifting. The blue frames around the objects indicate the tracked poses.

## Figures and Tables

**Table 1:** Summary of the objects in our initial database release.
